# Comparative assessment of growth performance and meat quality in Water Hyacinth and antibiotic (growth promoter) supplemented broilers

**DOI:** 10.1016/j.psj.2025.105105

**Published:** 2025-03-28

**Authors:** Afrina Mustari, Md. Iqramul Haque, Samia Rashid, Md Sabbir Khan, Mahabub Alam, Mohammad Alam Miah, Md. Abul Kalam Azad, Emdadul Hauqe Chowdhury

**Affiliations:** a Department of Physiology, Faculty of Veterinary Science, Bangladesh Agricultural University, Mymensigh, 2202, Bangladesh; b Department of Animal Science, Faculty of Animal Husbandry, Bangladesh Agricultural University, Mymensigh, 2202, Bangladesh; c Department of Pathology, Faculty of Veterinary Science, Bangladesh Agricultural University, Mymensigh, 2202, Bangladesh

**Keywords:** Broiler, Ciprofloxacin, Growth promoter, Meat quality, Water Hyacinth

## Abstract

The use of growth promoters in broiler feed is a common practice to enhance feed efficiency and accelerate growth rates. Ciprofloxacin, a widely used antibiotic in poultry farming, promotes growth and disease prevention but raises concerns about antibiotic residues in meat and the development of antibiotic resistance. This study examines the dietary effects of WH and ciprofloxacin on broiler growth rate, feed conversion ratio (FCR), meat quality, and muscle histology. Ninety broilers were randomly assigned to one of three groups: a control group, a WH-supplemented group (2.5% of daily feed), and a ciprofloxacin (Cipro) group (8 mg/kg body weight). Feed consumption and body weight were monitored daily, and after sacrifice, breast muscles were collected for meat quality assessment and histopathological analysis. WH supplementation significantly improved (*P* < 0.05) body weight gain, feed conversion ratio (FCR), and meat quality in broilers. Compared to control and ciprofloxacin groups, WH-treated broilers exhibited lower cooking and drip loss (*P* < 0.05), higher water-holding capacity (*P* < 0.01), and reduced shear force value (*P* < 0.05), indicating improved texture. Ciprofloxacin treatment resulted in reduced redness (*P* < 0.001) and increased yellowness (*P* < 0.01), while lightness remained unchanged across groups. Histological analysis revealed greater perimysium thickness and intermuscular adipose infiltration in WH-treated broilers, suggesting enhanced muscle structure. WH supplementation presents a safer, sustainable alternative to antibiotics in broiler production.

## Introduction

Poultry meat is a widely accepted and preferred source of animal protein in Bangladesh, with broiler meat gaining increasing popularity (Rahman et al., 2007). Chicken is a key, affordable contributor to animal protein in human diets (Rahman et al., 2017). The extensive use of antibiotics in poultry farming, primarily to enhance growth and reduce disease, has been prevalent since 1951 (Castanon, 2007), though rising feed costs and disease outbreaks threaten profitability (Engberg et al., 2000; Page and Gautier, 2012). Ciprofloxacin is such a representative fluoroquinolone antibiotic with a vast antimicrobial spectrum and high bactericidal activity (Bauernfeind and Petermüller, 1983; Fass, 1983; Wise et al., 1984; Chin et al., 1984) which is commonly used by farmers as a growth promoter (Huang et al., 2024; Haque et al., 2017; Khan et al., 2019). Even though, the use of growth promoters (GPs) has ensured production but also sacrificed the quality of production creating ubiquitous public health risks (Sultana et al., 2023). Such widespread use of antibiotics for promoting growth could easily contribute to the already alarming pool of antibiotic-resistant bacteria. Residues of antibiotics in meat and other products can directly harm consumer's health and at the same time, an indirect effect could be their role in producing resistance in several human pathogens (Emborg et al., 2004). Consequently, the European Union has banned the use of antibiotic growth promoters in poultry diets since 2006 (Janardhana et al., 2009), thus motivating researchers to seek effective alternatives such as probiotics, prebiotics, herbal products, marine natural products, and organic acids (Ayasan, 2013). Therefore, an organic production system needs to be established in no time to achieve sustainability in the growth of this sector (Sultana et al., 2023). Production scientists are evaluating different medicinal plants and their active components as feasible alternatives to antibiotics to ensure health and production for many years (Chowdhury et al., 2018; Arif et al., 2022). The beneficial impacts of these plants or plant products are mostly brought about by their antioxidant, antimicrobial, appetite-stimulating, and immune-boosting capacities (Eevuri and Putturu, 2013). Phytogenic growth promoters are ideal for poultry because they are natural, residue-free, eco-friendly and have no side effects. The phytogenic growth promoters showed antimicrobial, antiparasitic, insecticidal, antifungal, antiviral and antitoxic effects (Yitbarek, 2015).

Water hyacinth (***Eichhornia crassipes***) (WH) a medicinal herb, belonging to the family Pontederiaceae, is such a perennial aquatic plant that is indigenous to tropical and sub-tropical countries (Carnevali et al., 2017; Hoseinifar et al., 2018). It is a cosmopolitan invasive aquatic plant that can tolerate a wide range of environmental conditions such as temperature, humidity, illumination, pH, salinity, wind, current and drought. In Bangladesh, a huge amount of WH are produced due to a large number of rivers, ponds, lakes and other water reservoirs. Even though some treated it as the world's worst aquatic weed plant (Indulekha et al., 2019), these plants are utilized for animal consumption because of their availability and nutrient value (Simpson and Sanderson, 2002) and low cost of production. Nowadays, the administration of such medicinal herbs is increasing as an alternative practice for antibiotics and other chemotherapeutics (Carnevali et al., 2017; Hoseinifar et al., 2018). The proximate analysis of WH revealed that water hyacinth is constituted of 50 % protein and 33 % carbohydrates, while the remaining nutrients are made up of fat, ash, and fibre (Adeyemi and Osubor, 2016). Moreover, water hyacinth leaf protein concentrate (WHPLC) can be used as a food supplement due to the high protein content and sufficient content of xanthophylls, carotenes, unsaturated fats, starch, and essential minerals such as calcium, phosphorus, and iron (Kateregga and Sterner, 2007). Seventeen out of twenty amino acids were detected in the water weed without asparagine, glutamine, and tryptophan (Adeyemi and Osubor, 2016). In addition, the leaves contain plentiful water-soluble vitamins B1, B2, B3, B5, B6, and B12 and fat-soluble vitamins E and A (GG Monsod Jr - US Patent 4 et al., 1981). Furthermore, it has been reported that there are antioxidants in water hyacinth leaves and the protein concentrate made from them may be used as a food ingredient (Bodo et al., 2004). However, the literature was inadequate in terms of profound details and further studies on the extraction of these water weeds in edible form. There were a few reports available on meat yield and quality traits of fast-growing broilers and fed on water Hyacinth-based diets by varying ingredients and nutrient composition worldwide (Dumaup and Ampode, 2020; Suthama, 2004; Al-Aboudi and Hamodi, 2023). There is a clear gap in investigating the effects of WH inclusion instead of ciprofloxacin, a commonly used growth promoter of fast-growing broilers. Therefore, the study was conducted to evaluate the growth response of WH-fed broilers, and examine the meat quality of broilers for safer, cost-effective alternatives to antibiotics, ultimately preventing residue and antibiotic resistance in humans and satisfying consumers’ preference for meat traits.

## Materials and methods

### Ethical Standard

This study was carried out following institutional ethical standards and authorized by the Animal Welfare and Experimentation Ethics Committee, Bangladesh Agricultural University (BAU), Bangladesh [Authorization no. - AWEEC/BAU/2024 (38)]. The study was carried out in the poultry shed of the Department of Physiology, BAU, Mymensingh-2202, from February to July 2024.

### Experimental animals and chemicals

The study was carried out in the Department of Physiology at Bangladesh Agricultural University, Mymensingh-2202. A total of 90 day-old-broiler chicks were selected to evaluate the comparative effects of water hyacinth and ciprofloxacin. The chicks were raised in an open-house system for 30 days, with routine vaccinations administered according to schedule. Both feed and water were provided *ad-libitum* throughout the experiment.

### Preparation of Water Hyacinth Meal (WHM)

Whole plants of water hyacinth were collected fresh from the lakes of Bangladesh Agricultural University, Mymensingh-2202. Collected plants were washed and scrutinized to remove all unwanted matters (lake debris, leather wrappings, and other extraneous materials), cut manually, and sundried for three days to reach 10 % moisture content. Dried plants were ground using an attrition mill and sieved through a 1 mm sieve to produce water hyacinth meal and stored in large plastic containers with tight-fitting lids until needed. Proximate composition (Table 1) (moisture, ash, protein, and fibre) was evaluated following the official AOAC methods 2005.

**Table 1:** Proximate analysis of water hyacinth.

Name	%Moisture	%Crude lipid	%Crude Protein	% Ash	% Crude fiber	% Carbohydrate
**Leaf**	83.15	0.77	0.93	2.48	5.14	7.53
**Trunk**	92.10	0.64	0.95	1.10	4.33	0.88

### Diets and experimental protocol

The experiment was conducted using 90 broilers, randomly divided into three groups of 30 birds each. The 1^st^ group served as the vehicle control and received only the standard poultry feed. The WH group was supplemented with daily doses of water hyacinth meal (2.5 %) in addition to the feed. The Cipro group was administered ciprofloxacin (8 mg/kg body weight) for three consecutive days, along with water. The trial lasted for 30 days. At the end of the study, on the 30th day, the broilers were sacrificed using manual cervical dislocation. The birds were immediately dissected, and breast muscle samples were collected for further analysis.

### Measurements of live body weight

The live body weight of each bird in each group was measured daily using the digital balance and the total body weight gain was computed by subtracting the initial live body weight from the final live body weight.

Bodyweightgain=Finalbodyweight−Initialbodyweight

### Feed Conversion Ratio (FCR)

Feed consumption refers to the amount of feed consumed by the birds in a given time. Feed intake was calculated by subtracting the amount of feed supplied to the birds from the amount of feed remaining at the end of each feeding period. The FCR was determined by dividing the total feed consumed by the total body weight gain which denotes unit of feed per unit of body weight gain.

FCR=Totalfeedconsumedbythebirds(g)/Totalbodyweightgain(g)

### Meat color test

The surface color of the collected broiler meat sample was measured by a CR-400 Chroma Meter (Minolta Co., Osaka, Japan). Two to three-cm thick deboned meat samples were used to avoid background influence. The evaluation was done on the posterior surface of the skinless breast meat. The meat color was expressed in terms of CIE values where L*, a*, and b* indicate the lightness, redness, and yellowness of the meat samples, respectively. Hue angle [tan^1^ (b*/a*)] and Saturation index (SI) = {(a*^2^ + b*^2^)^1/2^} were then computed for each sample to study the change in color of the meat samples (Islam et al., 2022).

### Breast meat pH

The pH measurement of the samples was carried out with a pH HI 99163 pH meter (HANNA instruments. Inc. Highland Industrial Park, USA). The pH meter reading was set to 7.00 by dipping its head into a neutral buffer solution at 24 °C temperature. pH reading was taken from three different regions of each meat sample, and then the mean value was determined (Islam et al., 2022).

### Measurement of Water Holding Capacity (WHC) of meat

The WHC of the meat samples was measured using centrifugal force. For this, 1 g of breast and thigh meat was weighed (W_0_) from each sample and then chopped with a meat chopper. Then the chopped meat was loaded in a 1.5 ml Eppendorf tube and the total weight of the sample along with the tube was measured and recorded as W_1_. The tubes were then placed inside the centrifuge machine. Centrifugal force was applied through 10,000 RCF (Relative Centrifugal Force) at 48C temperature for 10 min. Then the supernatant fluid was removed properly by micropipette. The weight of the sample along with the tube was again measured and recorded as W_2_. Finally, the WHC was computed using the following formula and the data was carefully recorded (Islam et al., 2022)

WHC(%)=[1−{(W1−W2)/W0}]×100

### Measurement of cooking loss

The meat samples reserved for cooking loss were weighted, and then taken in the double-layer polythene bag. Then they were broiled on Farberware Open Hearth electrical broiler (Farberware, Bronx, NY). Samples were turned every 4 min during broiling until an internal temperature of 71 °C was reached. The internal temperature was monitored by a digital thermometer (Model 31308-KF, Atkins Tech. Inc., Gainesville, FL) placed in the approximate geometric center of each sample. Afterwards, samples were taken from the water bath and were spread for 10 min for surface drying. After 10 min, when surface drying was completed samples were weighed again. Then cooking loss was measured by using the following formula and the data was recorded carefully.

$$Cookingloss\left(\%\right)=\frac{\left(Sampleweightbeforecooking-Sampleweightaftercooking\right)}{\left(Sampleweightbeforecooking\right)}\times 100$$

### Measurement of drip loss

At 24 hrs. after post-mortem, the drip losses of breast muscles were measured. Approximately 25g (wet weight) of regular-shaped muscle was cut from the breast at the same position for each sample and then weighed, which was recorded as initial weight. The sample was placed in an airtight box by hanging on a string and stored at 4 °C in a refrigerator. After 24 hrs, all the samples were taken from the freezer and reweighed (final weight) by using a digital balance. The drip loss was calculated by using the following formula and the data was recorded carefully.

$$Driploss\left(\%\right)=\frac{\left(Initialweightofthesample-finalweightofthesample\right)}{\left(Initialweightofthesample\right)}\times 100$$

### Measurement of Shear Force Value (SFV)

The meat samples were taken in a double-layer polythene bag. Then they were broiled on Farberware Open Hearth electrical broiler (Farberware, Bronx, NY). .Samples were turned every 4 min during broiling until an internal temperature of 71 °C was reached. The internal temperature was monitored by a digital thermometer (Model 31308-KF, Atkins Tech. Inc., Gainesville, FL) placed in the approximate geometric center of each sample. Cooked steaks were cooled to room temperature (≈20 °C) before five to six 1.27-cm cores were sheared once, perpendicular to the muscle fibre orientation on a Warner-Bratzler shear machine (G-R Elec. Mfg. Co, Manhattan, KS). This machine measures the maximum force required to cut across the muscle fibres. The Warner-Bratzler shear values will be expressed as Newton of shear force (N) required. An average shear force was calculated and recorded for each meat sample.

### Histomorphological Study

The formalin-fixed muscle samples were processed, sectioned, and stained with Hematoxylin and Eosin stain (H&E) for histological investigation as per standard protocol (Suvarna et al., 2018) in collaboration with the Department of Pathology, Bangladesh Agricultural University. The stained slides were viewed using a Trinocular light microscope (Olympus, Japan).

### Statistical analysis

All data were collected and recorded in Microsoft Excel 2019, then transferred to GraphPad Prism 9.0 for statistical analysis. A two-way repeated measures ANOVA, followed by an appropriate post hoc test, was applied to evaluate weekly weight gain and feed conversion ratio (FCR). For other parameters, a one-way ANOVA was used, followed by Bonferroni's multiple comparisons test. Statistical significance was considered at *P < 0.05, **P < 0.01, and ***P < 0.001.

## Results and discussions

### Effect of water hyacinth and ciprofloxacin on growth performance and FCR

**Fig. 1:**
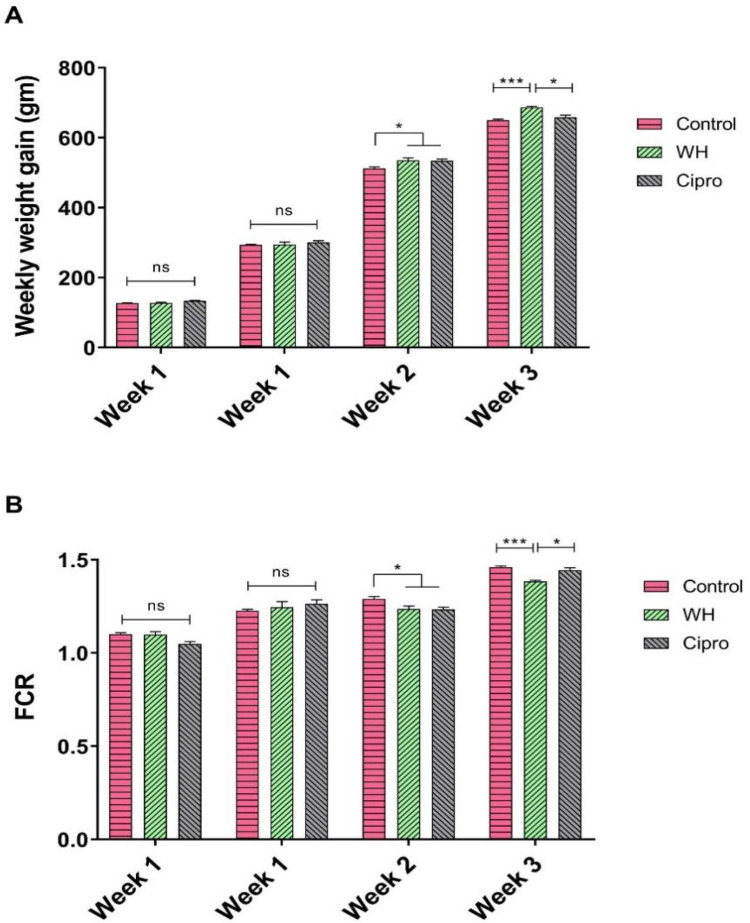
Impact of WH and Ciprofloxacin supplementation on growth performance in broilers. (A) Weekly weight gain per bird across different treatment groups. (B) Weekly feed conversion ratio (FCR) across different treatment groups. Data are presented as mean ± SEM, with * indicating *P* < 0.05, ** indicating *P* <0.01, and *** indicating *P* < 0.001.

Weekly body weight gain was consistently monitored and analyzed across all groups. During the first two weeks, no statistically significant differences were observed in body weight gain (Fig. 1A) between the control group and either the WH or Cipro groups (*P* > 0.05). However, at 3^rd^ week, a marked increase in body weight gain was evident in both the WH and Cipro groups compared to the control (*P* < 0.05). At week four, both treatment groups exhibited significantly higher gains in body weight (Fig. 1A) relative to the control group (*P* < 0.001), with the WH group showing a more pronounced increase compared to the Cipro group (*P* < 0.05). These results suggest that the dietary interventions began to exert measurable effects on weight gain after the initial two weeks, with the WH supplementation proving especially effective in promoting growth.

For the feed conversion ratio (FCR), no statistically significant differences were observed across the groups (Fig. 1B) during the first two weeks (*P* > 0.05). However, by the end of week three, both the WH and Cipro groups demonstrated significant improvements in FCR (Fig. 1B) compared to the control (*P* < 0.05), indicating enhanced feed efficiency. In week four, the WH group exhibited the lowest FCR among all groups (Fig. 1B), significantly lower than both the control (*P* < 0.001) and Cipro (*P* < 0.05), further highlighting the superior feed efficiency associated with WH supplementation.

During the initial two weeks, the absence of significant differences in body weight gain between the treatment groups and the control suggests that both WH and Cipro require a latency period before their biological effects on growth become evident. However, by the third week, both treatment groups displayed significantly higher weight gains than the control group, with this effect being even more pronounced by the fourth week, especially in the WH-supplemented group.

A previous study on broilers demonstrated that incorporating a 2.5 % WH-supplemented meal into their feed positively influenced body weight gain (Ampode et al., 2020). The mechanism underlying the enhanced weight gain in broilers supplemented with WH may be linked to the nutritional composition of Water Hyacinth, which is known to contain a variety of bioactive compounds such as polyphenols, flavonoids, and essential fatty acids (Carnevali et al., 2017; Hoseinifar et al., 2018). These components could contribute to improved nutrient absorption and metabolism, thereby supporting accelerated growth. Additionally, the high fibre content of WH might promote a healthy gut micro biota, enhancing digestion and nutrient assimilation. Studies have shown that dietary fibre, when provided in optimal amounts, can serve as a substrate for beneficial gut microbes, leading to the production of short-chain fatty acids (SCFAs) which play a key role in energy metabolism and growth (Cronin et al., 2021; Singh and Kim, 2021; Fu et al., 2022).

The observed improvements in FCR in both the WH and Cipro groups by the third week reflect more efficient feed utilization, likely as a result of the enhanced nutrient bioavailability associated with both supplements. WH, in particular, led to a significantly lower FCR by the fourth week, indicating superior feed efficiency relative to both the control and Cipro groups. This suggests that WH supplementation not only promotes greater weight gain but also allows broilers to convert feed into body mass more efficiently. The increased efficiency may be attributed to the antioxidant properties of the bioactive compounds in WH (Bodo et al., 2004; Noufal et al., 2023), which could reduce oxidative stress and improve cellular energy metabolism. Oxidative stress is known to impair growth performance in poultry by compromising cellular functions and increasing metabolic costs (Oke et al., 2024). Therefore, the antioxidant effects of WH could help mitigate these negative impacts, enabling better feed conversion.

In contrast, the effects of Cipro, while beneficial in improving both weight gain and FCR, were less pronounced compared to WH. The antibiotic properties of Cipro may have contributed to a more favorable gut environment by suppressing pathogenic bacteria, but its impact on growth and feed efficiency appears less multifaceted compared to WH. Antibiotic supplementation often improves growth by reducing the microbial competition for nutrients (Miyakawa et al., 2024); however, the broader nutritional and bioactive profile of WH seems to confer additional advantages that extend beyond mere pathogen control.

### Effect of water hyacinth and Ciprofloxacin on meat quality pH, Redness (a*), Yellowness (b*), and Lightness (L*) of Meat

**Fig. 2:**
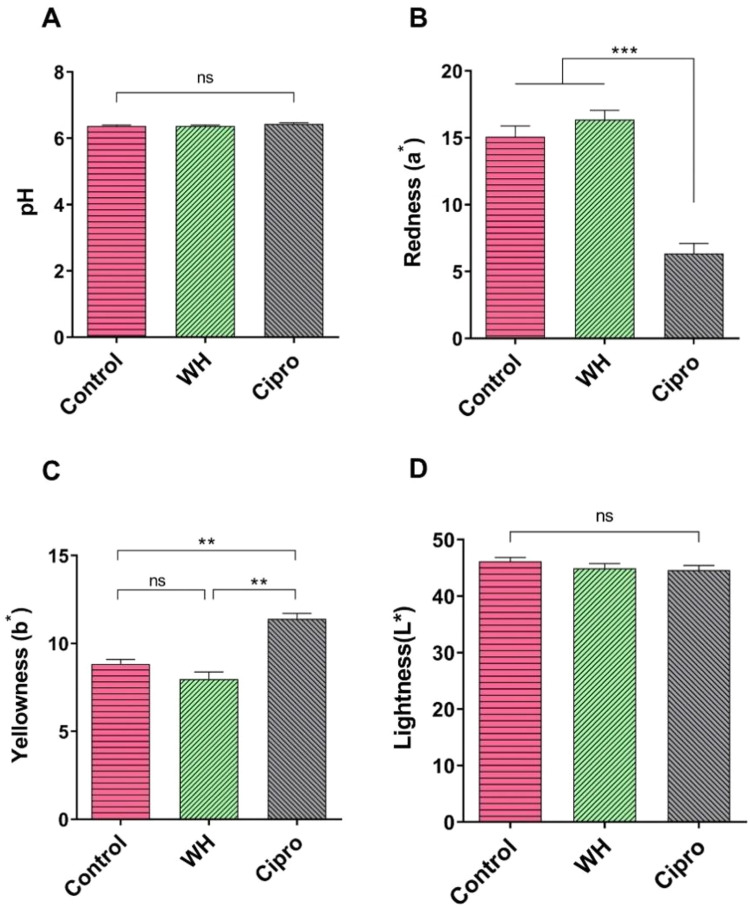
Effect of WH and Ciprofloxacin supplementation on meat pH and color profile in broilers across different experimental groups. (A) Meat pH in different treatment groups. (B) Meat redness (C) Meat yellowness (D) Meat lightness across experimental groups. Data are presented as mean ± SEM. Statistical analysis was performed using one-way ANOVA followed by Bonferroni's multiple comparison test. ** *P* < 0.01, and *** *P* < 0.001.

We measured the pH values of representative samples from the control, WH, and Cipro groups to assess potential differences in meat acidity. Across all groups, the pH levels were comparable (Fig. 2A), with no statistically significant differences observed.

In contrast, the colorimetric properties of the breast meat (Fig. 2B-D), assessed in terms of redness (a*), yellowness (b*), and lightness (L*), showed notable variations among different groups. The redness values in the WH-supplemented group were similar to those of the control, whereas the Cipro group exhibited a significant reduction in redness (*P* < 0.001) compared to both the control and WH groups (Fig. 2B). In terms of yellowness, the Cipro-supplemented samples displayed significantly higher values (*P* < 0.01) than those from the control and WH groups (Fig. 2C). Lightness, on the other hand, remained consistent across all three groups (Fig. 2D), showing no significant variation.

The pH levels across the control, WH, and Cipro groups in our study showed no statistically significant differences. Meat pH is an important determinant of post-mortem muscle biochemistry, affecting water-holding capacity, tenderness, and shelf life (Mir et al., 2017).The comparable pH values across the groups in our study suggest that neither WH nor Cipro had a significant impact on muscle acidity, consistent with findings from a previous study (Costa et al., 2007). Typically, muscle pH is influenced by glycogen content and post-slaughter glycolysis, where high pH values indicate less glycogen depletion and lower lactic acid accumulation (Baldi et al., 2020). Since pH remained uniform across the groups, it can be inferred that the dietary interventions did not alter the glycolytic metabolism of the broilers.

However, the colour profiles of the breast meat exhibited distinct differences, which may reflect the biochemical effects of the dietary treatments on muscle composition. The redness (a*) values in the WH group remained similar to those of the control, while the Cipro group demonstrated a significant reduction in redness (*P* < 0.001). Redness in meat is primarily associated with myoglobin content and its oxidative state, which is indicative of oxygen-carrying capacity in muscle. The reduction in redness in the Cipro group could be linked to alterations in muscle oxidation or a reduction in myoglobin levels, potentially due to Cipro's antimicrobial action. Antibiotic use can disrupt the gut microbiota, which plays a role in nutrient absorption and muscle oxygenation processes. A decrease in redness could imply less oxidative stability, a quality concern, as paler meat is generally less appealing to consumers and may indicate compromised shelf life or freshness.

In contrast, WH supplementation did not affect the redness values, suggesting that WH might help maintain normal muscle oxygenation and myoglobin levels, potentially due to the antioxidant properties of bioactive compounds such as flavonoids and polyphenols present in WH. Antioxidants can protect myoglobin from oxidative degradation, thereby preserving the natural redness of the meat. This maintenance of redness aligns with better overall meat quality, as redness is a preferred characteristic for consumer acceptance, reflecting freshness and a favourable nutrient profile.

Meat color is influenced by pH, with darker shades linked to higher pH (Wideman et al., 2016). Our study showed treated broilers displayed a more reddish-yellow hue, indicating improved meat quality, consistent with results from broilers supplemented with marine red seaweed (Halymenia palmata) (Balasubramanian et al., 2021). Li et al. (2020) found that eucalyptus leaf polyphenol extract in feed raised both a* values (redness) and myoglobin content due to antioxidant properties. Consumers generally prefer the pink hue typical of chicken breast meat (Choo et al., 2014). (Bungsrisawat et al., 2018) similarly observed increased a* and b* values in native chicken, aligning with trends in our study. The WH group's stable yellowness values may result from Water Hyacinth's phytochemicals, which protect muscle lipids from oxidative damage, thus preserving meat quality.

Interestingly, the lightness (L*) values remained consistent across all groups, showing no significant variation. Lightness in meat is usually correlated with water-holding capacity and structural integrity of muscle fibres. Since no differences were observed, it suggests that neither WH nor Cipro supplementation had a significant impact on the meat's moisture content or protein structure, which aligns with the stable pH levels observed across all groups.

### WHC, cooking loss, drip loss, and SFV of breast meat

**Fig. 3:**
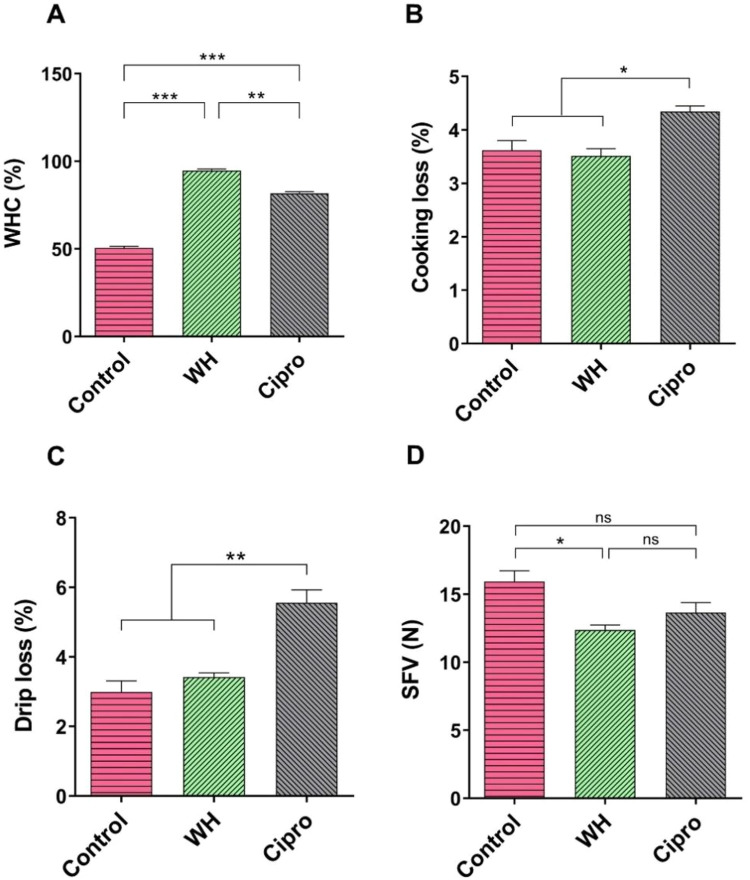
Effect of WH and Ciprofloxacin supplementation on meat water-holding capacity (WHC), cooking loss, drip loss, and shear force value (SFV) in broilers across different experimental groups. (A) WHC (%) in different treatment groups. (B) Cooking loss. (C) Drip loss. (D) SFV across experimental groups. Data are presented as mean ± SEM. Statistical analysis was conducted using one-way ANOVA followed by Bonferroni's multiple comparison test. * *P* <0.05, ** *P* < 0.01, and *** *P* < 0.001.

We next evaluated the water-holding capacity (WHC) of the representative meat samples (Fig. 3A), revealing a significant increase (*P* < 0.01) in WHC in the WH-treated group compared to both the control and Ciprofloxacin (Cipro) groups. As WHC is critical in determining the visual appeal, weight loss, cooking yield, and sensory attributes, we further analyzed the cooking loss in these groups (Fig. 3B). The WH-treated meat exhibited similar cooking loss to the control group, with both showing significantly lower cooking loss (*P* < 0.05) than the Cipro group (Fig. 3B).

Regarding drip loss, the use of Ciprofloxacin led to a marked increase in drip loss (*P* < 0.01) compared to both the control and WH-treated groups (Fig. 3C). Additionally, we assessed the shear force value (SFV) as a measure of meat tenderness. The WH-treated group demonstrated the lowest SFV among the groups (Fig. 3D), indicating significantly more tender meat (*P* < 0.05) than the control group. In contrast, the Cipro-treated group had SFV values comparable to the control (Fig. 3D), suggesting similar tenderness levels between these two groups.

The significantly higher WHC observed in the WH-treated group (*P* < 0.01) compared to both the control and Cipro groups suggests that WH supplementation positively affects the meat's ability to retain water during storage and cooking. WHC is crucial because it directly impacts the visual appeal of meat and its juiciness (Yang et al., 2011; Warner, 2017), which are important for consumer satisfaction. Water-holding capacity (WHC) is vital as it impacts meat's visual appeal and juiciness, both key to consumer satisfaction (Yang et al., 2011; Warner, 2017). Higher WHC improves moisture retention, reducing cooking-related weight loss and preventing dry, tough meat. It also affects carcass yield, influencing profitability (Dai et al., 2009). The enhanced WHC in the WH-treated group may stem from Water Hyacinth's fibre and bioactive compounds, which improve muscle cell integrity and water retention, aligning with findings in other species that dietary fibres strengthen muscle tissues to hold more water (Zheng et al., 2023; Jha and Mishra, 2021). Despite higher WHC, the WH-treated group's cooking loss remained similar to the control but was significantly lower than the Cipro group (*P* < 0.05), suggesting optimal water retention and yield during cooking. The Cipro group's higher cooking loss implies increased moisture and nutrient loss, likely reducing meat's juiciness and quality.

Notably, drip loss, an indicator of post-slaughter moisture retention, was also significantly higher in the Cipro group (*P* < 0.01). High drip loss typically leads to drier meat with reduced visual appeal, which can be detrimental to consumer acceptance. The marked increase in drip loss in the Cipro group may indicate compromised muscle cell structure, potentially due to altered muscle metabolism or increased oxidative stress (Wang et al., 2017). This aligns with earlier findings of reduced redness and increased yellowness in the Cipro group, which may reflect oxidative damage to muscle tissues (Wang et al., 2017). In contrast, the WH-treated group exhibited significantly lower drip loss, suggesting that WH supplementation helps maintain better muscle integrity, preventing excessive moisture loss during storage. This improvement in WHC and reduced drip loss likely enhances the juiciness and freshness of the meat, making it more attractive to consumers (Mir et al., 2017; Swatland, 2008).

Our study demonstrated that the use of antibiotics increases cooking loss, which aligns with the findings of Costa et al. (2007) and Hamid et al. (2019), who reported higher cooking loss in birds treated with tylosin and other antibiotic growth promoters. In contrast, the inclusion of the non-antibiotic growth promoter WH in this study significantly reduced cooking loss, thereby minimizing nutrient loss. These results are consistent with the observations of Balasubramanian et al. (2021), who reported similar effects when supplementing broilers with red seaweed.

The shear force value (SFV), a critical measure of meat tenderness (Lonergan et al., 2003), revealed another notable effect of WH supplementation. The WH-treated group had significantly lower SFV (*P* < 0.05) than the control, indicating more tender meat. Tenderness is a key quality attribute influencing consumer preference, and lower SFV values are associated with softer, more palatable meat. The reduced SFV observed in WH group was similar to the findings reported by Cui et al., where meat from the selected line had a significantly lower SFV than that in the control line, implying increased tenderness (Cui et al., 2022). The improved tenderness in the WH group could be linked to the bioactive compounds in Water Hyacinth, such as polyphenols and flavonoids (Shanab et al., 2010), which have been shown to reduce muscle fibre rigidity and improve protein degradation during post-mortem aging. By potentially modulating muscle protein breakdown and reducing connective tissue toughness, WH may contribute to a more desirable texture in broiler meat. The control and Cipro groups had similar SFV values, suggesting that Ciprofloxacin did not enhance tenderness and maintained a texture similar to the unsupplemented broilers.

### Histomorphology of Muscle

**Fig. 4:**
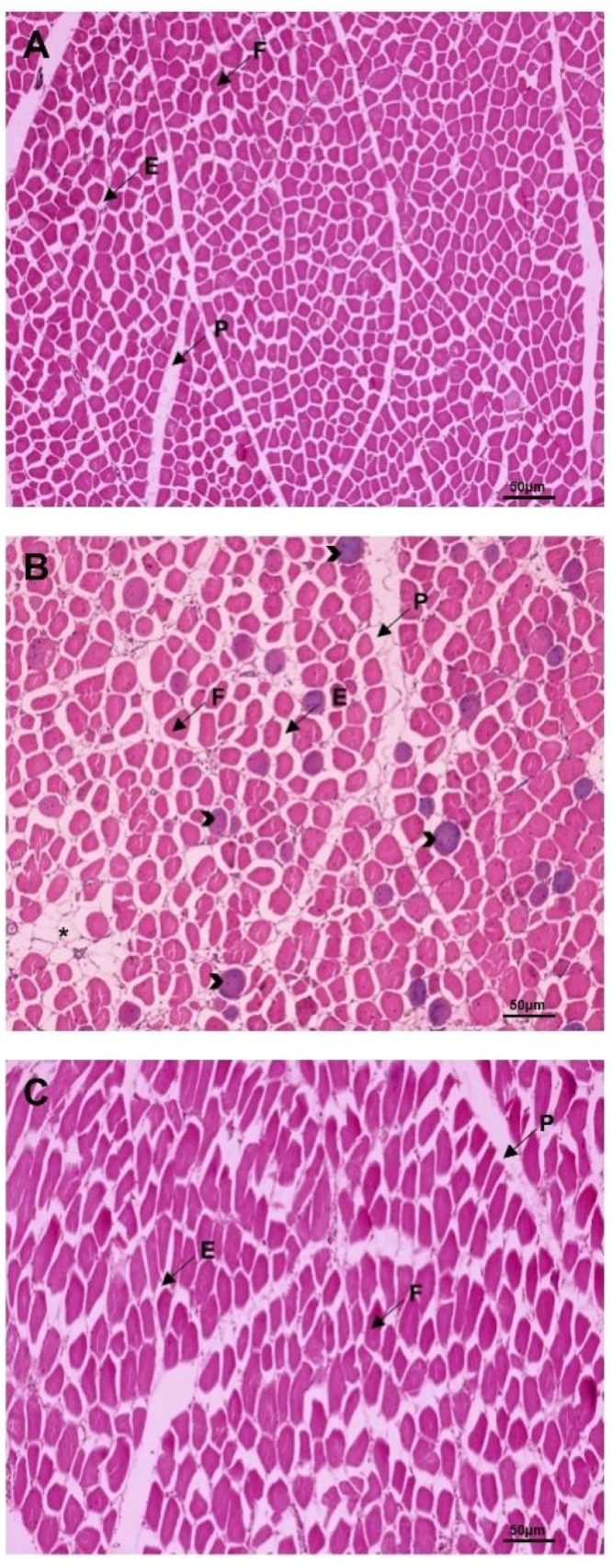
Effects of Water Hyacinth and Ciprofloxacin on muscle samples across different groups. Photomicrographs of muscle tissue from birds in the control group (A), WH-treated group (B), and Ciprofloxacin-treated group (C), at 100x magnification. Scale bar = 50 μm. F = muscle fiber; P = perimysium thickness; E = endomysium thickness; (*) = adipose tissue infiltration; (>) = enlarged muscle bundle.

The histomorphological evaluation of muscle tissue across the groups (Fig. 4) revealed significant structural changes in the WH-treated broilers (Fig. 4B), particularly an increase in perimysium thickness and inter-muscular adipose tissue infiltration compared to the control (Fig. 4A) and Cipro groups (Fig. 4C). The perimysium, a connective tissue surrounding muscle fibre bundles, plays a crucial role in determining meat texture. Its increased thickness in the WH group is closely associated with enhanced tenderness, as thicker perimysium has been shown to reduce the resistance to cutting, resulting in lower shear force values and more tender meat (An et al., 2010; Garcia et al., 2010). This structural modification aligns with the observed improvements in water-holding capacity (WHC), drip loss, and cooking loss, suggesting that thicker perimysium and adipose infiltration contribute to better water retention and fat distribution, which are key factors in overall meat quality and tenderness.

The infiltration of adipose tissue within the muscle further supports the improvements in tenderness and juiciness in WH-supplemented broilers. Evenly distributed fat can disrupt the muscle fibre matrix, facilitating easier breakdown during cooking, and thereby enhancing both tenderness and moisture retention. This anatomical modification also likely explains the increased WHC observed in the WH group, as fat serves as a barrier to moisture loss during cooking, contributing to the sensory qualities of the meat, including its flavor and tenderness.

These findings are consistent with prior research, including An et al. (2010), who documented a positive correlation between perimysium thickness and tenderness, as well as an inverse relationship with myofibre density and endomysium thickness. The observed increase in perimysium thickness and myofibre size in WH-treated broilers likely reduces myofibre density, thereby facilitating muscle fibre separation and contributing to lower shear force values, aligning with previous studies on the role of perimysium in meat texture (Brooks and Savell, 2004). Collagen, a predominant component of connective tissue within the perimysium, influences meat tenderness. Although collagen is typically associated with toughness, its interaction with adipose tissue in WH-treated broilers appears to enhance rather than diminish tenderness. This outcome is consistent with findings by Nakamura et al. (2003), who demonstrated that connective tissue alterations substantially impact meat tenderness and quality. Additionally, the structural changes observed in WH-supplemented broilers—specifically, the thickened perimysium and increased adipose infiltration—likely underlie the observed improvements in water-holding capacity, reductions in drip and cooking losses, and enhanced tenderness. These histomorphological modifications support the premise that dietary interventions, such as WH supplementation, can significantly influence muscle structure and, consequently, meat quality.

## Conclusions

The findings of this study demonstrate that supplementing broiler diets with 2.5 % WH significantly improves body weight gain and feed conversion ratio (FCR). WH supplementation also enhances the color profile and overall meat quality, resulting in better visual appeal and tenderness compared to both control groups and those treated with antibiotic growth promoters. Additionally, WH-treated broilers exhibited increased perimysium thickness and adipose infiltration, indicating structural modifications that contribute to superior meat quality. Therefore, WH supplementation presents a safer and more cost-effective alternative to antibiotic growth promoters, addressing concerns related to antibiotic residues and resistance in humans, while meeting consumer preferences for desirable meat characteristics. Future research should focus on elucidating the underlying mechanisms or isolating specific bioactive compounds in WH responsible for these growth-promoting and meat-enhancing effects in broilers.

## Disclosures

The authors declare that they have no known competing financial interests or personal relationships that could have appeared to influence the work reported in this paper.

## Declaration of competing interest

The authors declare that they have no known competing financial interests or personal relationships that could have appeared to influence the work reported in this paper.

## Appendix Supplementary materials

Image, application 1
