# Supplementary material for: Comparative assessment of growth performance and meat quality in Water Hyacinth and antibiotic (growth promoter) supplemented broilers
Source: Poult Sci. 2025 Mar 28;104(6):105105. doi: 10.1016/j.psj.2025.105105 (PMC12002923; doi:10.1016/j.psj.2025.105105)
Supplement: Supplementary file 1 [file mmc1.docx]

**Figure 1. Impact of WH and Ciprofloxacin supplementation on growth performance in broilers. (A) Weekly weight gain per bird across different treatment groups. (B) Weekly feed conversion ratio (FCR) across different treatment groups. Data are presented as mean ± SEM, with * indicating P < 0.05, ** indicating P <0.01, and *** indicating P < 0.001.**

**Figure 2. Effect of WH and Ciprofloxacin supplementation on meat pH and color profile in broilers across different experimental groups. (A) Meat pH in different treatment groups. (B) Meat redness (C) Meat yellowness (D) Meat lightness across experimental groups. Data are presented as mean ± SEM. Statistical analysis was performed using one-way ANOVA followed by Bonferroni's multiple comparison test. ** P < 0.01, and *** P < 0.001.**

**Figure 3. Effect of WH and Ciprofloxacin supplementation on meat water-holding capacity (WHC), cooking loss, drip loss, and shear force value (SFV) in broilers across different experimental groups. (A) WHC (%) in different treatment groups. (B) Cooking loss. (C) Drip loss. (D) SFV across experimental groups. Data are presented as mean ± SEM. Statistical analysis was conducted using one-way ANOVA followed by Bonferroni's multiple comparison test. * P <0.05, ** P < 0.01, and *** P < 0.001.**

**Figure 4. Effects of Water Hyacinth and Ciprofloxacin on muscle samples across different groups. Photomicrographs of muscle tissue from birds in the control group (A), WH-treated group (B), and Ciprofloxacin-treated group (C), at 100x magnification. Scale bar = 50 μm. F = muscle fiber; P = perimysium thickness; E = endomysium thickness; (*) = adipose tissue infiltration; () = enlarged muscle bundle.**
